# Transcriptome Analysis of Retinoic Acid-Inducible Gene I Overexpression Reveals the Potential Genes for Autophagy-Related Negative Regulation

**DOI:** 10.3390/cells11132009

**Published:** 2022-06-23

**Authors:** Shaotang Ye, Chen Tan, Xiaoyun Yang, Ji Wang, Qi Li, Liang Xu, Zhen Wang, Jianwei Mao, Jingyu Wang, Kui Cheng, Aolei Chen, Pei Zhou, Shoujun Li

**Affiliations:** 1 College of Veterinary Medicine, South China Agricultural University, Guangzhou 510642, China; yeshaotang@stu.scau.edu.cn (S.Y.); wangjiwj@stu.scau.edu.cn (J.W.); liqi1111@stu.scau.edu.cn (Q.L.); liangxu@stu.scau.edu.cn (L.X.); wangzhenwz@stu.scau.edu.cn (Z.W.); maojianwei@stu.scau.edu.cn (J.M.); wangjingyu@stu.scau.edu.cn (J.W.); 20203073013@stu.scau.edu.cn (K.C.); chenolay@scau.edu.cn (A.C.); zhoupei@scau.edu.cn (P.Z.); 2 Guangdong Provincial Key Laboratory of Prevention and Control for Severe Clinical Animal Diseases, Guangzhou 510642, China; 3 Guangdong Technological Engineering Research Center for Pet, Guangzhou 510642, China; 4 Lanzhou Veterinary Research Institute, Chinese Academy of Agricultural Science, Lanzhou 730046, China; chen.tan@doct.uliege.be; 5 Molecular and Cellular Epigenetics (GIGA) and Molecular Biology (TERRA), University of Liege, 4000 Liege, Belgium; 6 Zhaoqing Institute of Biotechnology Co., Ltd., Zhaoqing 526000, China; xiaoyunyangzqib@outlook.com

**Keywords:** RIG-I, overexpression, autophagy, negative regulation, transcriptome analysis

## Abstract

Retinoic acid-inducible gene I (RIG-I) serves as an essential viral RNA sensor for innate immune. The activation of the RIG-I-like receptors (RLRs) pathway triggers many regulations for the outcome of type I interferon, including ubiquitination, dephosphorylation, ISGylation, and autophagy. However, the autophagy-related regulation of RIG-I is still not fully understood. To investigate the potentially unknown genes related to autophagy-related regulation of RIG-I, we firstly confirm the induction of autophagy derived by overexpression of RIG-I. Furthermore, the autophagy inducer and inhibitor drugs were used in different assays. The results showed autophagy could control the activation of RLRs pathway and expression of exogenous RIG-I. In addition, we carried out the transcriptome analysis of overexpression of RIG-I in vitro. Differentially expressed genes (DEGs) in GO and KEGG signaling pathways enrichment provided a newly complex network. Finally, the validation of qPCR indicated that the DEGs PTPN22, PRKN, OTUD7B, and SIRT2 were correlated to the negative regulation of excessive expression of RIG-I. Taken together, our study contributed new insights into a more comprehensive understanding of the regulation of excessive expression of RIG-I. It provided the potential candidate genes for autophagy-related negative regulation for further investigation.

## 1. Introduction

Retinoic acid-inducible gene I (RIG-I) is a member of the RIG-I-like receptors (RLRs) family. It consists of the N-terminal caspase activation and recrement domain (CARD), DExD box domain, helicase domain, and the C-terminal regulatory domain [1,2,3,4]. RIG-I can sense the non-self RNA in the cytoplasm, like viral RNA. When non-self RNA is detected, RIG-I changes its original self-inhibitory conformation into activated conformation [5,6]. Mitochondrial antiviral signaling protein (MAVS) subsequently interacts with RIG-I and transduces the signaling to IRF-3/IRF-7 and NF-κB. These transcriptional factors promote the outcome of type I interferon (IFN) [7,8].

An appropriate amount of IFN performs a strong antiviral effect on the different viruses. However, overexpressed IFN is triggered by the auto-immune system or overacted response to viral infection sometimes [9,10,11]. This overaction will be incredibly harmful to host cells. To keep the cellular homeostasis, cells develop many precise regulation systems for the RLRs pathway to control the innate immune response, including ubiquitination [12], dephosphorylation [13], and ISGylation [14,15]. Autophagy is a conservative biological process that breaks down and recycles old, damaged, or abnormal proteins and other substances in a lysosome-dependent manner [16]. Recently, several studies indicated that autophagy plays an essential role in the negative regulation of innate immune response. RLRs pathway is also regulated by the accurate autophagy process [17]. However, many potential mechanisms are still not fully understood or discovered. These unknown autophagy-related regulations may need further screening or multi-omics techniques to unearth the potential genes.

This study aimed to confirm the ability of autophagy to negatively regulate RIG-I protein and investigate the potential genes in negative regulation of RIG-I overexpression by transcriptome analysis.

## 2. Materials and Methods

### 2.1. Cells, Virus, Plasmid, Reagents, and Antibodies

Madin–Darby canine kidney (MDCK) cells and human embryonic kidney (HEK) 293T cells were cultured at 37 °C and 5% (*v*/*v*) CO_2_ in Dulbecco’s modified Eagle medium (DMEM; Biological Industries, Kibbutz Beit-Haemek, Israel) with 10% fetal bovine serum (Biological Industries). Canine influenza virus (A/canine/Guangdong/02/2011) was propagated in specific pathogen-free chick embryos. Plasmids used in this research was listed Supplementary Table S1. Specific RIG-I agonist, 5′ triphosphate hairpin RNA (3p-hpRNA) (InvivoGen, San Diego, CA, USA), was used for RIG-I activation. Small interfering RNA (siRNA) was synthesized (RiboBio, Guangzhou, China) and the sequence was listed in Supplementary Table S2. Antibodies used in this study were listed in Supplementary Table S3.

### 2.2. Cell Seeding, Transfection, RNA Extraction, and cDNA Synthesis

Cells (1 × 10^5^, 2 × 10^6^ and 6 × 10^6^) were seeded in 24-, 12-, and 6-well plates. Cells were grown to 80% confluence and transfected with plasmids, empty vector, RIG-I agonist, or siRNA. Total RNA was extracted from cells with Simply P Total RNA Extraction Kit (Bioer Technology, Hangzhou, China) according to the manufacturer’s instructions. RNA samples were stored at −80 °C. Then, 1000 ng cDNA was prepared from total RNA using the HiScript III 1st Strand cDNA Synthesis Kit (Vazyme, Nanjing, China) and used for qPCR.

### 2.3. RNA Sequencing and Data Analysis

RNA samples were isolated from HEK 293T cells transfected with 2500 ng Flag-RIG-I or 2500 ng empty vectors. cDNA libraries were produced by the NEBNext Ultra Directional RNA Library Prep Kit (New England Biolabs, NEB, Ipswich, MA, USA), NEBNext Poly(A)mRNA Magnetic Isolation Module (NEB), NEBNext Multiplex Oligos (NEB) according to the manufacturer’s instructions. The purified and enriched cDNA libraries were quantified by Agilent2200 (Agilent Technologies, Santa Clara, CA, USA), and the tagged cDNA libraries were pooled in equal ratio and used for 150 bp paired-end sequencing in a single lane of the Illumina HiSeq X Ten (Illumina, San Diego, CA, USA). After removing the adaptor sequences and low-quality reads, the clean reads were used for subsequent analysis, and the unique reads were used to identify differentially expressed genes (DEGs) by EdgeR (standard, log2(FC) > 1 or <−1 and FDR < 0.05).

### 2.4. Functional Gene Ontology and Kyoto Encyclopedia of Genes and Genomes Signaling Pathways Analysis

To analyze the DEGs, we used Fisher’s exact test for the Gene Ontology (GO) analysis in three parts, including molecular function, cellular component, and biological process. Additionally, the significantly differential GO terms (*p* < 0.01) were performed as GO trees. In addition, we also used Fisher’s exact test for the Kyoto Encyclopedia of Genes and Genomes (KEGG) signaling pathways analysis, and the significantly differential pathway terms (*p* < 0.05) were performed as path-act-network.

### 2.5. Fluorescence Analysis

To determine the subcellular localization of LC3 or P62 proteins, HEK 293T cells were transfected with Flag-RIG-I plasmid or empty vector along with LC3-GFP-mCherry or P62-mCherry plasmid, respectively, when cells reached 60% confluence. Cells were washed three times with PBS and then fixed in cold 4% paraformaldehyde at room temperature for 10 min. Nuclei were stained with 4′,6-diamidino-2-phenylindole (Beyotime, Shanghai, China) for 5 min. The fluorescence signal was observed with the confocal laser scanning microscope, Leica SP8 TCS (Leica, Wetzlar, Germany).

### 2.6. Dual-Luciferase Assay

HEK 293T cells (1 × 10^5^) were seeded in a 24-well plate. Cells were grown to 80% confluence and co-transfected with 250 ng/well of reporter plasmid and 20 ng/well of pRL-TK plasmid along with 500 ng of 3p-hpRNA, or empty vector (as control). After 24 h, the cells were lysed, and the luciferase activity in lysates was measured with the Dual-Luciferase Reporter Assay Kit (Vazyme) according to the manufacturer’s instructions. The internal Renilla luciferase control normalized the luciferase activity.

### 2.7. Real-Time qPCR (qPCR)

qPCR was carried out on a LightCycler 480 (Roche, Basel, Switzerland) with ChamQ SYBR qPCR Master Mix (Vazyme), and the qPCR program followed the manufacturer’s protocol. The relative mRNA expression level was calculated with the 2^−∆∆Ct^ method and indicated a fold change compared to the control group. Primer sets used for qPCR were designed based on published sequences and are listed in Supplementary Table S4.

### 2.8. Western Blotting

The cells were lysed using Cell lysis buffer for Western and IP (Beyotime). Lysates were collected and centrifuged at 15,000 rpm for 15 min. A total of 30–50 μg of each sample was separated by SDS-PAGE and transferred to a PVDF membrane blocked for at least 10 min with QuickBlock Blocking Buffer for Western Blot (Beyotime) at room temperature and then incubated overnight at 4 °C with primary antibodies. After washing with PBST, secondary antibodies were incubated for 1 h at room temperature. Protein bands were visualized by Odyssey Sa (Li-cor, Lincoln, NE, USA).

### 2.9. Autophagy-Related Treatment

HEK 293T cells (1 × 10^5^ and 6 × 10^6^) were seeded in 24- and 6-well plates for fluorescence analysis, dual-luciferase assay and Western blotting, respectively. Cells were grown to 80% confluence and transfected with plasmids or 3p-hpRNA. After transfection for 12 or 24 h, cells were subsequently treated with rapamycin (1 μM, acting as an autophagy inducer), MG132 (20 μM, high concentration, acting as an autophagy inducer), Ly294002 (20 μM, acting as an autophagy inhibitor), and Chloroquine (50 μM, acting as an autophagy inhibitor) for 12 h.

### 2.10. Statistical Analysis

All data were analyzed by the unpaired Student’s *t*-test using Prism v9.3 (GraphPad Software, San Diego, CA, USA). (mean ± SD, * *p* < 0.05, ** *p* < 0.01, *** *p* < 0.001).

## 3. Results

### 3.1. Excessive Expression of Canine RIG-I Triggered Unknown Negative Regulations

**Figure 1:**
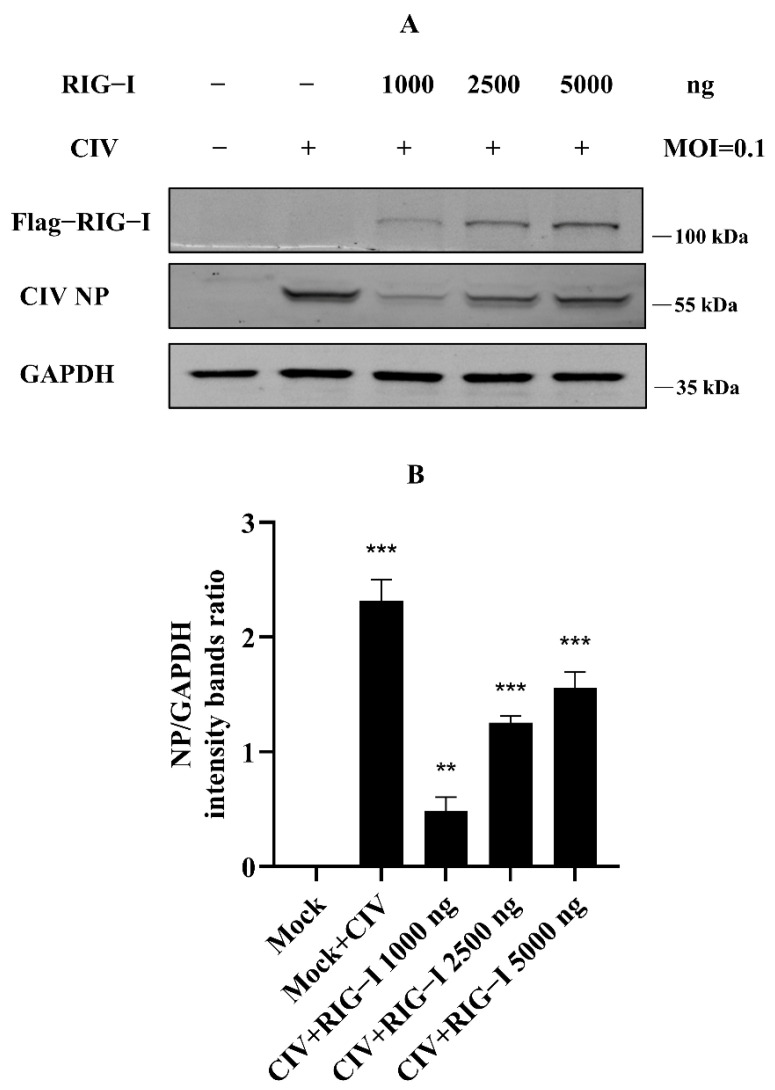
Excessive expression of canine RIG-I (transfection dose more than 2500 ng) weakened the antiviral effect against H3N2 CIV. (**A**) Flag-tagged RIG-I and viral NP was determined by Western blotting, and GAPDH was used as a loading control. (**B**) Protein band intensity of viral NP and GAPDH were used for Western blotting quantification analysis. Samples were analyzed in three independent experiments (** *p* < 0.01, *** *p* < 0.001).

Our previous study identified the antiviral effect of canine RIG-I against canine influenza virus (CIV), whereby overexpression of canine RIG-I could strongly suppress the CIV replication in MDCK and HEK 293T cells [18]. However, we found that more than 2500 ng canine RIG-I overexpression reduced the antiviral effect against CIV. To determine the authentication of the results of our previous study, we carried out the experiments in the same condition again, such that MDCK cells were transfected with canine RIG-I from 0 ng to 5000 ng, and cells were infected with CIV after 24 h. The whole protein of these cells was collected and isolated by Western blotting. The results showed that CIV NP protein significantly increased in the 2500 ng and 5000 ng canine RIG-I group (Figure 1A,B), similarly to the previous study. This suggested that the results of our experiments were reliable, and there were some unknown negative regulations to the excessive expression of RIG-I in cells. 

### 3.2. Cellular Autophagy Is Essential to the Regulation of Excessive RIG-I

**Figure 2:**
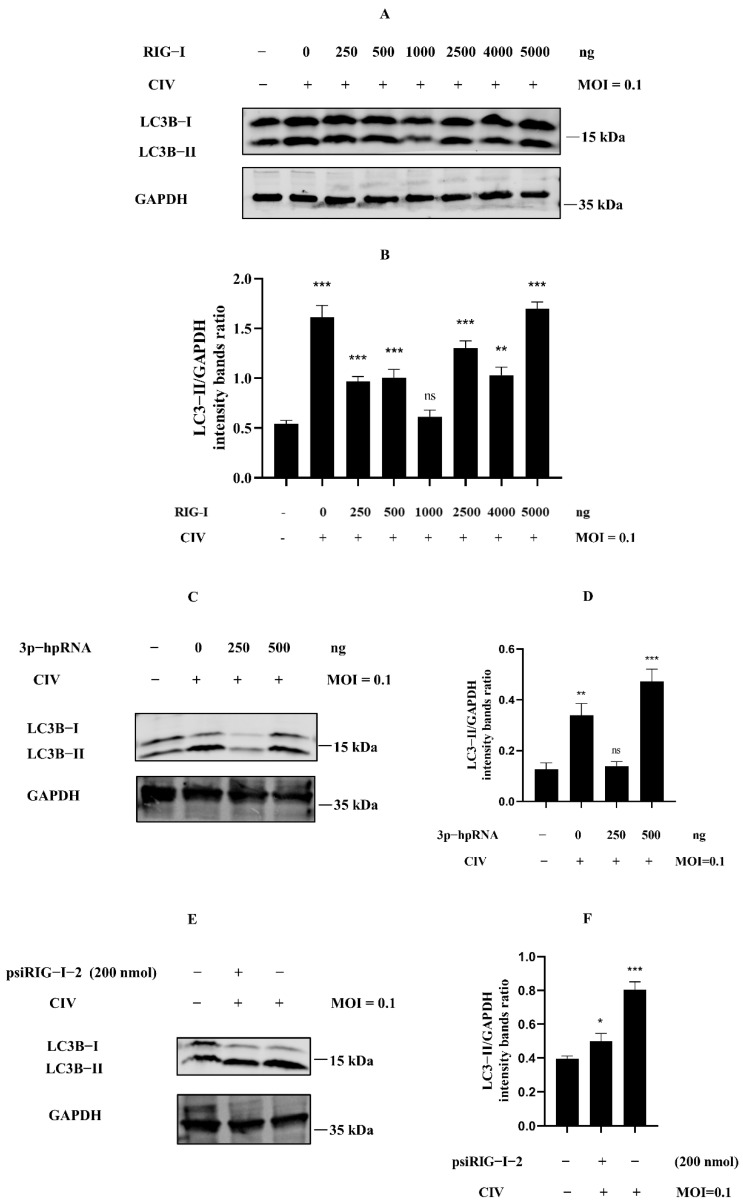
Excessive expression of RIG-I induced autophagy in CIV infection. (**A**) MDCK cells were transfected with indicated concentrations of Flag-RIG-I plasmid; after 24 h, cells were inoculated with H3N2 CIV at 0.1 multiplicity of infection (MOI), and the expression level of the endogenous LC3B was determined by Western blotting. (**C**,**E**) MDCK cells were transfected with indicated concentrations of RIG-I agonist (**C**) or psiRIG-I−2 (**E**); after 24 h, cells were inoculated with H3N2 CIV at 0.1 MOI, and the expression level of the endogenous LC3B was determined by Western blotting. GAPDH served as a protein sample loading control. (**B**,**D**,**F**) Protein band intensity of LC3B-II and GAPDH were used for Western blotting quantification analysis. Samples were analyzed in three independent experiments (ns indicated no significance, * p < 0.05, ** *p* < 0.01, *** *p* < 0.001).

Many studies about the negative regulation of RIG-I have indicated the relationship of the negative regulation for RIG-I, including ubiquitination, phosphorylation, ISGylation, and more post-translational modification. However, we still know relatively little about cellular autophagy serving for RIG-I regulation. To investigate whether cellular autophagy was involved in the phenomenon in Figure 1, we detected the expression level of LC3B protein in MDCK cells protein samples with the same treatments as our previous study [18]. The overexpression of RIG-I against H3N2 CIV experiments showed that the ratio of LC3B-II/GAPDH was relatively higher when the Flag-RIG-I plasmid dose for transfection was more than 2500 ng (Figure 2A,B). Interestingly, it showed the same trend in samples of MDCK cells transfected with a higher amount of 3p-hpRNA against H3N2 CIV (Figure 2C,D) and no siRNA of RIG-I against H3N2 CIV (Figure 2E,F). These results suggested that dose-dependent autophagy was involved in expressing canine RIG-I against the H3N2 CIV infection.

**Figure 3:**
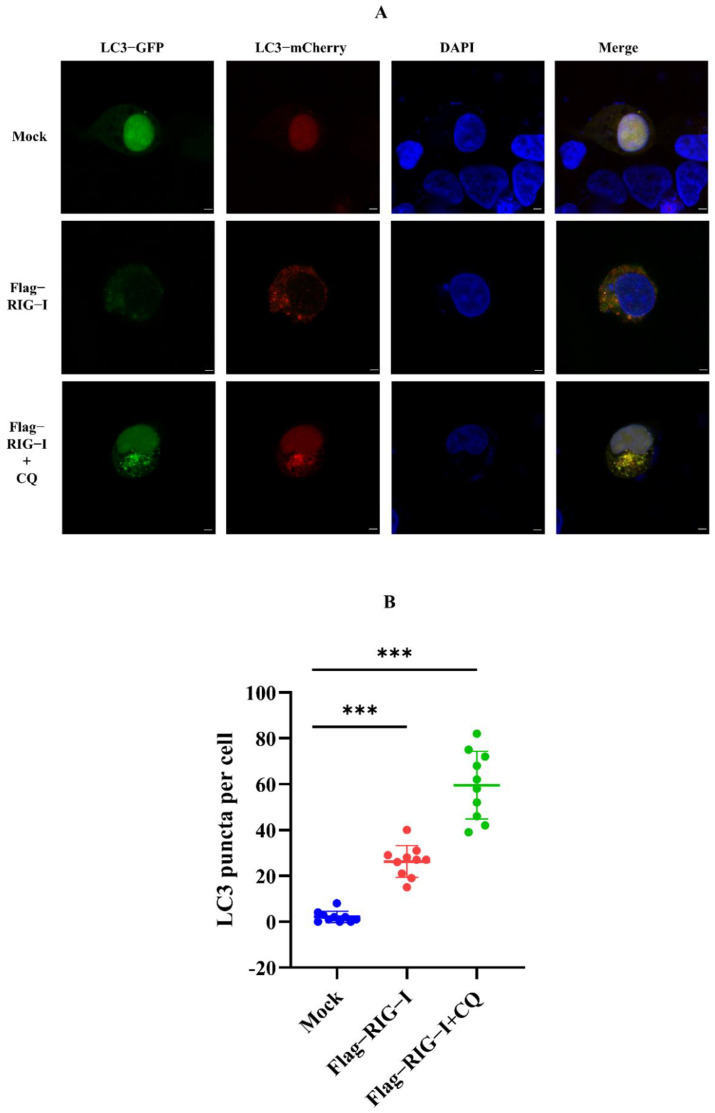
Observation of LC3 with overexpression of RIG-I in HEK 293T cells. (**A**) Fluorescence analysis for confocal observation of HEK293T cells transfected with Flag-RIG-I plasmid. Scale bar, 3 μm. (**B**) Numbers were counting of LC3-GFP-mCherry puncta formation in HEK 293T cells transfected with or without the Flag-RIG-I plasmid. (Data from 10 cells were shown, and bars represent the mean ± standard deviation, *** *p* < 0.001.)

**Figure 4:**
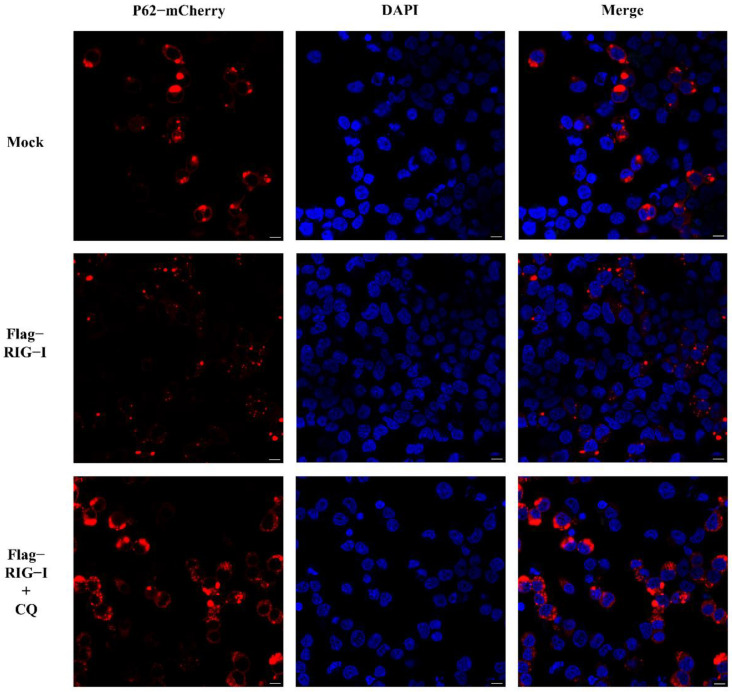
Observation of P62 with overexpression of RIG-I in HEK 293T cells with and without chloroquine treatment. Scale bar: 10 μm.

Nevertheless, whether CIV infection influenced this autophagy process remained unclear. To exclude this factor, we carried out an experiment without CIV infection. HEK 293T cells were co-transfected with 2500 ng of Flag-RIG-I and LC3-GFP-mCherry plasmids, or Flag-RIG-I and pCMV-mCherry-p62 plasmids, respectively. After 24 h transfection, cells were treated with and without chloroquine treatment. These cells were fixed and observed under a confocal microscope. The sample transfected with Flag-RIG-I showed a large number of representative autophagy-related LC3 puncta and chloroquine treatment significantly increased the LC3 puncta (Figure 3). In the observation of P62 protein, overexpression of Flag-RIG-I showed less P62 spots than mock group and chloroquine treatment group (Figure 4). This result suggested that excessive expression of RIG-I could induce autophagy without virus infection.

**Figure 5:**
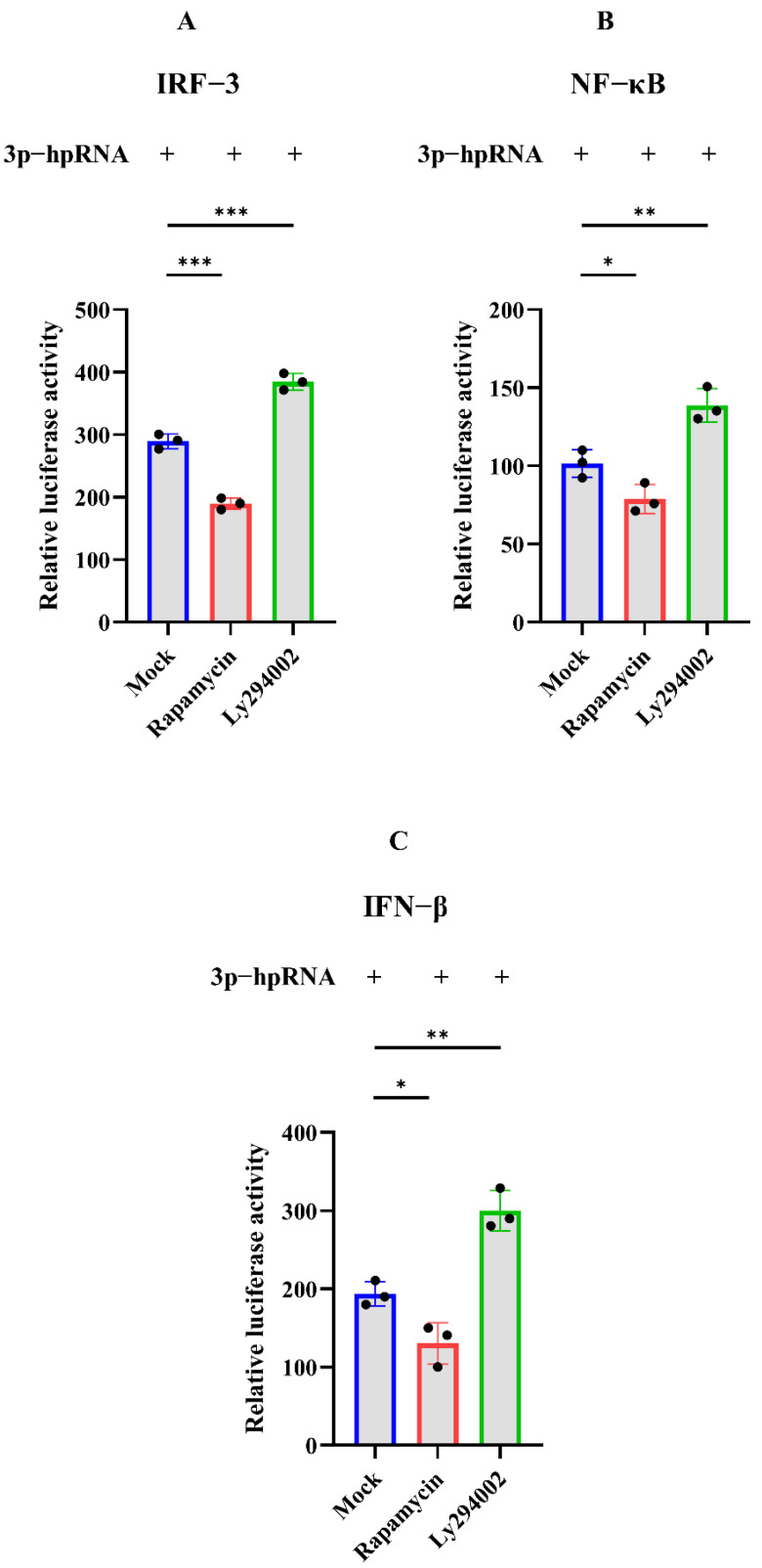
Dual-luciferase assay of 3p-hpRNA stimulation with autophagy-related treatment. HEK 293T cells were transfected with the reporter plasmids and 3p-hpRNA. After 12 h transfection, cells were treated with rapamycin (1 μM) and ly294002 (20 μM) for 12 h, respectively. (**A**) IRF-3 (**B**) NF-κB, and (**C**) IFN-β, promoter activity in the cells was evaluated for quantification analysis after 24 h transfection. Samples were analyzed in three independent experiments (* p < 0.05, ** *p* < 0.01, *** *p* < 0.001).

**Figure 6:**
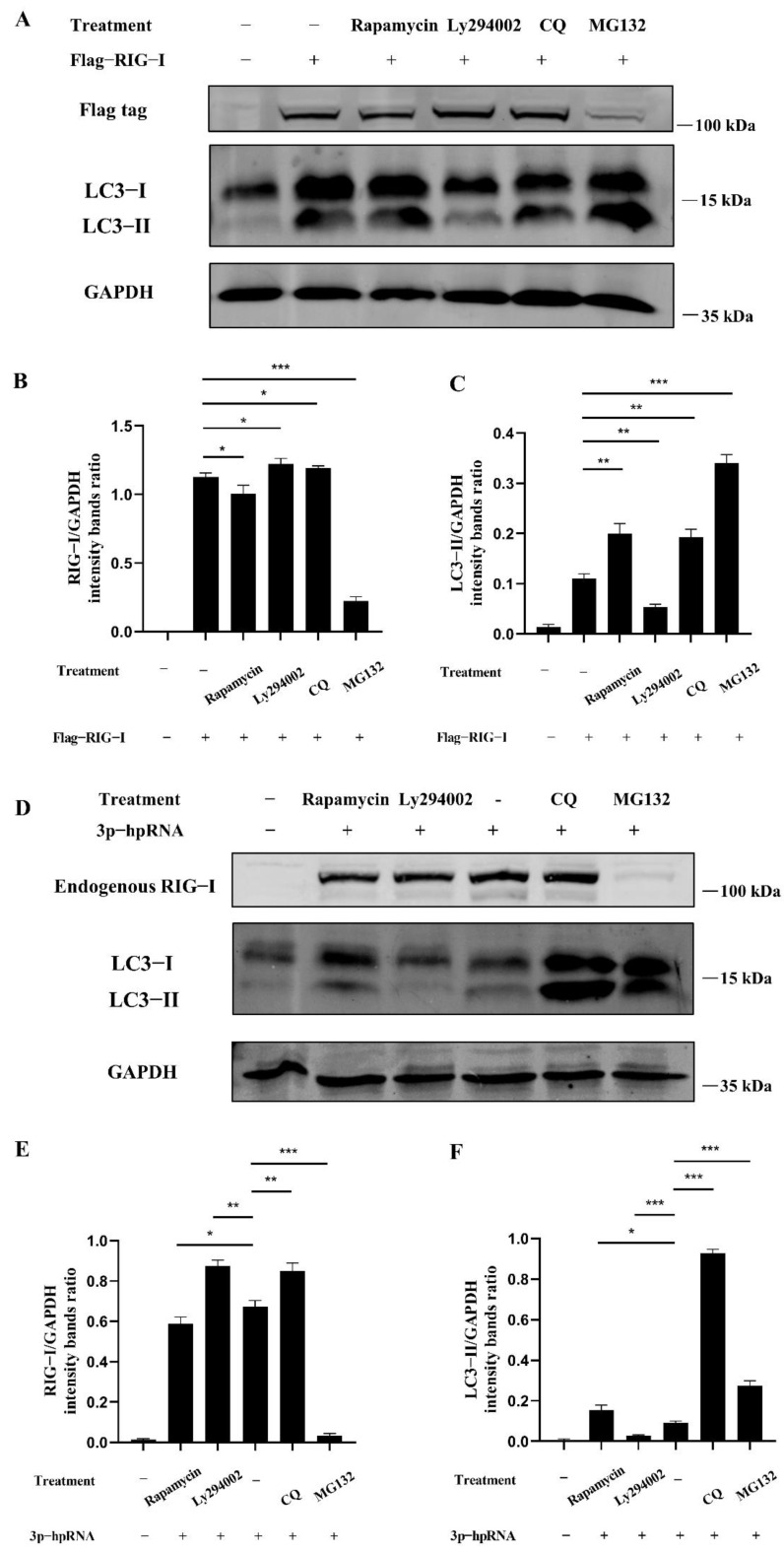
Autophagy regulates the signaling and expression of RIG-I. (**A**) HEK 293T cells were transfected with 2500 ng Flag-RIG-I. After 24 h transfection, cells were treated with rapamycin (1 μM), ly294002 (20 μM), chloroquine (50 μM), and MG132 (20 μM) for 12 h. (**D**) HEK 293T cells were stimulated with the 500 ng of 3p-hpRNA. After 24 h transfection, cells were treated with rapamycin (1 μM) and ly294002 (20 μM) for 12 h, respectively. (**B**,**E**) Protein band intensity of RIG-I and GAPDH were used for Western blotting quantification analysis. (**C**,**F**) Protein band intensity of LC3B-II and GAPDH were used for Western blotting quantification analysis. Samples were analyzed in three independent experiments (* p < 0.05, ** *p* < 0.01, *** *p* < 0.001).

The dual-luciferase and Western blotting assays were carried out to investigate whether autophagy acts as the negative regulation for excessive expression of RIG-I. In the dual-luciferase assays, HEK 293T cells were transfected with 3p-hpRNA. After 12 h transfection, cells were treated with rapamycin and ly294002 for 12 h, respectively. The agonist 3p-hpRNA was used for activation of RIG-I and its signaling. The results showed that rapamycin inhibited the activation of IRF-3, NF-κB, and IFN-β, while ly294002 enhanced the activation (Figure 5). In the Western blotting assays, 2500 ng of Flag-RIG-I plasmid was transfected into HEK 293T cells for 24 h, then cells were treated with rapamycin, ly294002, chloroquine, and MG132, respectively. The different treatments showed that rapamycin and high concentrations of MG132 decreased the expression of Flag-RIG-I while increasing the expression of Flag-RIG-I in ly294002 and chloroquine treatments (Figure 6A–C). In addition, 3p-hpRNA stimulation with autophagy-related treatment was also performed. The Western blotting results of endogenous RIG-I showed the same trend as the transfection of Flag-RIG-I with autophagy-related treatment (Figure 6D–F). Collectively, these results indicated that cellular autophagy played an essential role in the negative regulation of excessive expression of RIG-I.

### 3.3. Transcriptome Libraries Construction and Differentially Expressed Genes Analysis

**Figure 7:**
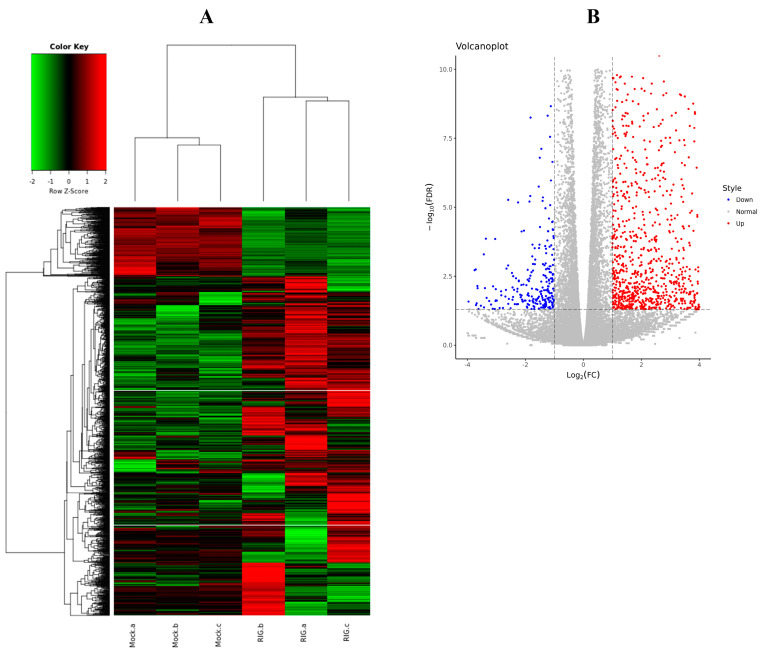
Differentially expressed genes (DEGs) analysis between Flag-RIG-I transfection group and mock transfection group. (**A**) Clustering analysis heatmap of DEGs. (**B**) Volcano plot visualization of DEGs.

To more comprehensively understand the inner cellular regulations triggered by the excessive expression of RIG-I, not only the negative regulation, six transcriptome libraries were prepared from HEK 293T cells transfected with Flag-RIG-I plasmid or empty vectors. Total RNA isolated from cells was used to establish the cDNA libraries. After filtering the adaptor sequences and low-quality reads, the whole clean reads were collected for subsequent analysis. Through comparative analysis according to the standard of logFC > 1 or <−1 and FDR < 0.05, 1666 differentially expressed genes (DEGs) were found in HEK 293T cells transfected with Flag-RIG-I, including 1384 up-regulated and 282 down-regulated mRNAs (Figure 7). All detailed DEGs were listed in Supplementary file 1.

### 3.4. Functional Enrichment of the Gene Ontology and Kyoto Encyclopedia of Genes and Genomes Signaling Pathways

To further analyze the DEGs, they were enriched according to the Gene Ontology (GO) and Kyoto Encyclopedia of Genes and Genomes (KEGG) signaling pathways, respectively.

**Figure 8:**
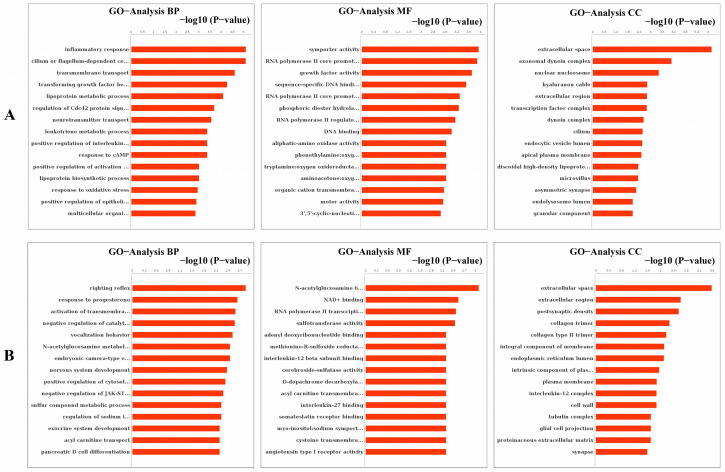
Analysis of GO enrichment in molecular function, cellular component, and biological process. (**A**) Analysis of up-regulated GO enrichment in three ontologies. (**B**) Analysis of down-regulated GO enrichment in three ontologies.

**Figure 9:**
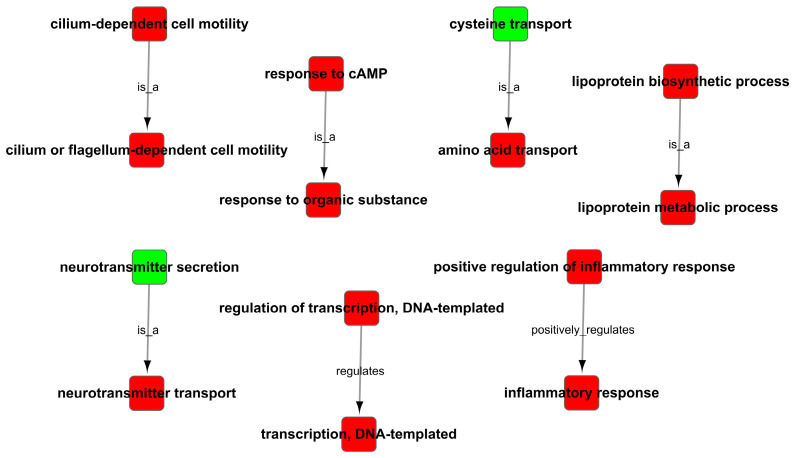
GO trees analysis of enrichment of GO terms.

As the results of the GO analysis involving biological processes, molecular functions, and cellular components, they showed that the DEGs were enriched in the inflammatory response, transmembrane transport, transforming growth factor-beta receptor signaling pathway, neurotransmitter transport, response to cAMP, response to oxidative stress and positive regulation of activation of JAK2 kinase activity in up-regulation (Figure 8A); the DEGs were enriched in right reflex, response to progesterone, tyrosine kinase activity, negative regulation of catalytic activity, nervous system development, and negative regulation of JAK-STAT cascade in down-regulation (Figure 8B). At the same time, the GO trees analysis of GO terms for the relationship between upstream and downstream regulation mainly indicated the positive regulation of inflammatory response, positive regulation of the lipoprotein-related metabolic process, positive regulation of response to cAMP and organic substance, positive regulation of transcription, and regulation of neurotransmitter (Figure 9).

**Figure 10:**
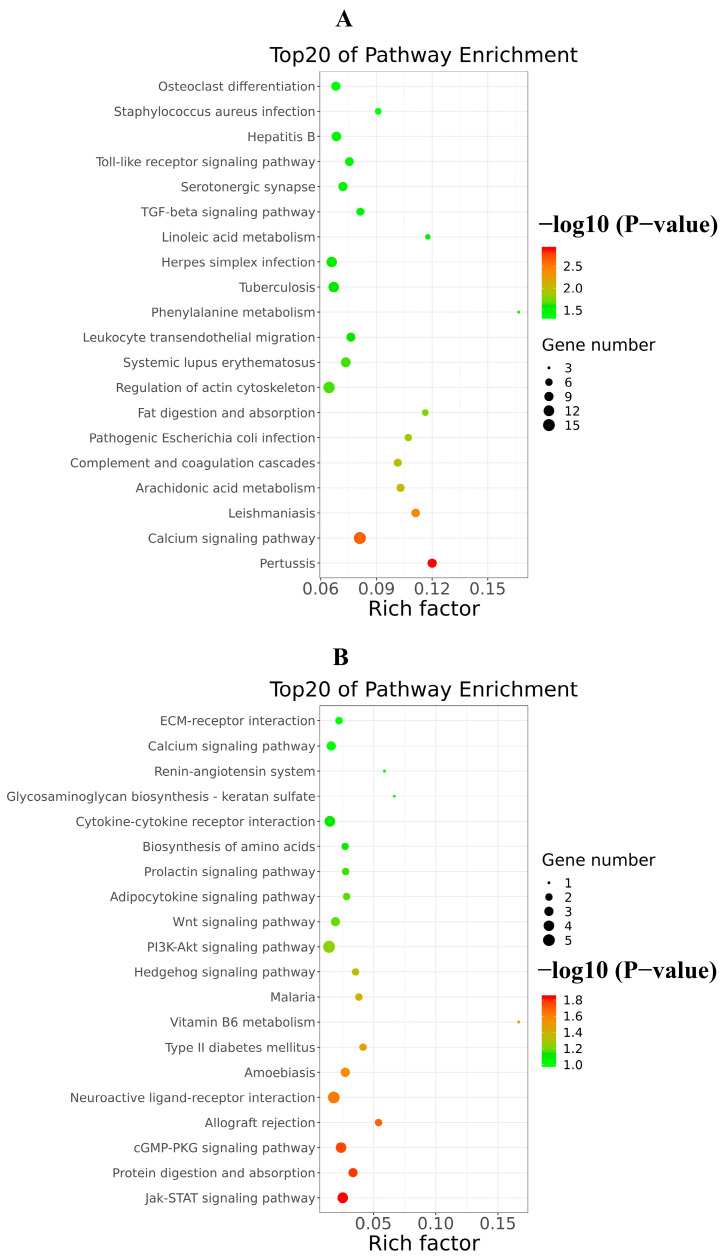
Top20 of KEGG signaling pathway enrichment of DEGs triggered by excessive RIG-I. (**A**) Up-regulated KEGG signaling pathway enrichment. (**B**) Down-regulated KEGG signaling pathway enrichment.

**Figure 11:**
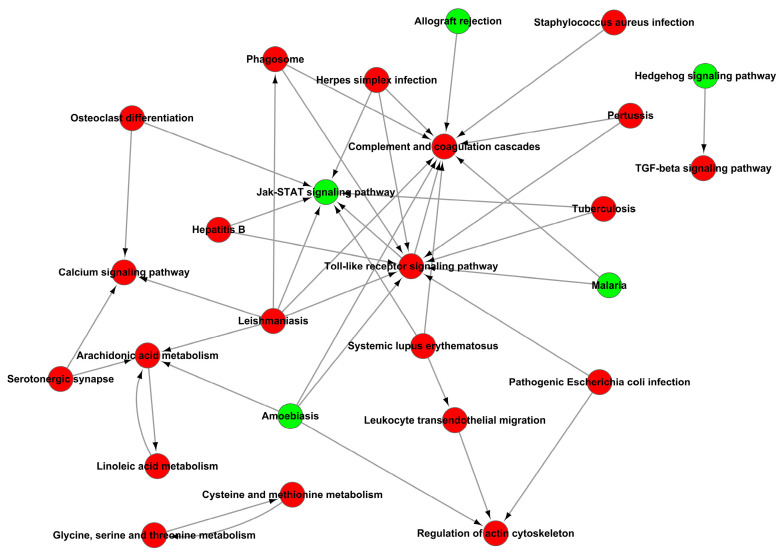
Path-act-network analysis of KEGG signaling pathway enrichment of DEGs.

In addition, the analysis of KEGG signaling pathways enrichment for excessive expression of RIG-I indicated several positive regulations of the pertussis pathway, calcium signaling pathway, leishmaniasis pathway, and arachidonic acid metabolism pathway (Figure 10A); it also indicated several negative regulations of the Jak-STAT signaling pathway, protein digestion and absorption pathway, cGMP-PKG signaling pathway, allograft rejection pathway, PI3K-AKT signaling pathway and neuroactive ligand-receptor interaction pathway (Figure 10B). There were many pathways influenced in cells transfected with RIG-I. To further clear up the relationship among these pathways, the path-act-network analysis of KEGG signaling pathway enrichment was performed. The results indicated complex crosstalk and several pathway centers, such as the Jak-STAT signaling pathway, arachidonic acid metabolism pathway, Toll-like receptor signaling pathway, and complement and coagulation cascades pathway (Figure 11). Taken together, these enrichments of GO and KEGG signaling pathways for transcriptional analyses greatly enriched our knowledge of regulation in HEK 293T cells with overexpression of RIG-I.

### 3.5. qPCR Validation of DEGs Related to Ubiquitination and Autophagy

**Figure 12:**
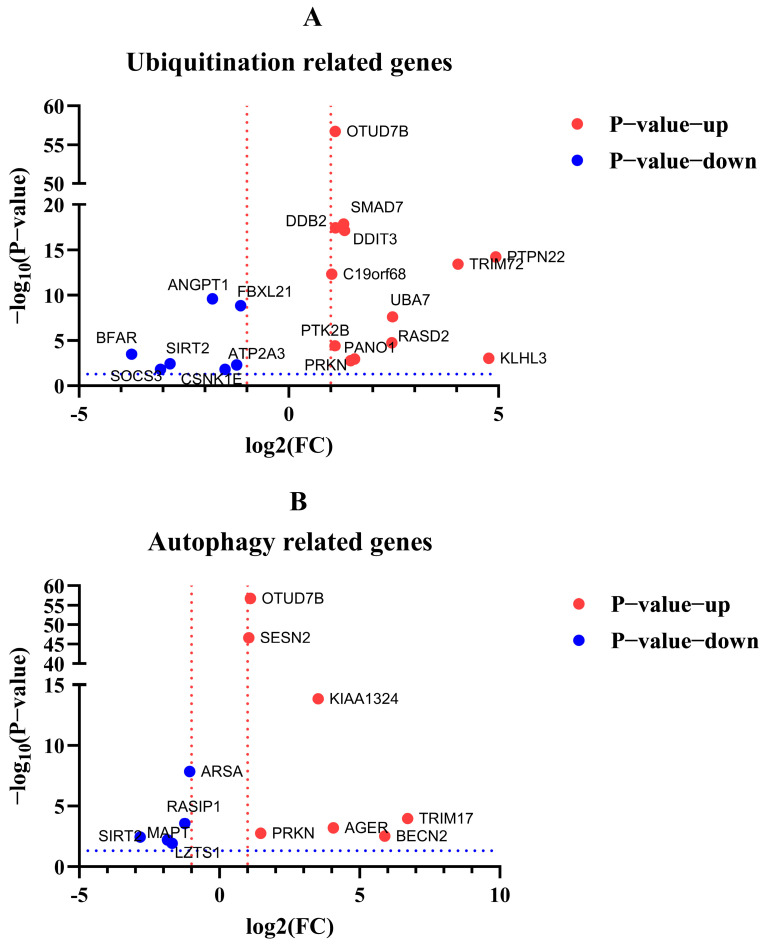
Volcano plot visualization of DEGs related to ubiquitination and autophagy. (**A**) DEGs related to ubiquitination. (**B**) DEGs related to autophagy. Red dotted line indicated the log_2_(FC) > 1 or < −1. Blue dotted line indicated the −log_2_(*p*-value) >−log_10_(0.05).

After the enrichment analysis of DEGs, we found that some genes about biological process ubiquitination and autophagy were also involved in regulation in cells with excessive expression of RIG-I. Here, we listed the related genes by volcano plot visualization (Figure 12). To unearth the potential genes for unknown negative regulation, qPCR was used to detect the mRNA level to confirm the differential fold change in the transcriptome of the original samples. The qPCR results suggested the PTPN22, UBA7, PRKN, SMAD7, TRIM72, OTUD7B, SIRT2, and BFAR genes related to ubiquitination or autophagy, were indeed regulated and consistent with the same trends in transcriptome when cells were overexpressed with RIG-I (Table 1). These genes may serve as the keys to previously mentioned autophagy-related negative regulation for excessive expression of RIG-I, and they need more validation and investigation in further related studies.

## 4. Discussion

Pathogen-associated molecular patterns (PAMPs) play an essential role in the innate immune system. RIG-I, which belongs to the RIG-I-like receptors (RLRs) family, acts as a mainly cytosolic viral RNA detector in eukaryotes [19,20,21]. After the recognition, RIG-I activates the RIG-I-like signaling pathway, which makes signaling transduction to promote the production of type I interferon (IFN) through IRF-3 and NF-κB [22,23,24]. The output of IFN activates the downstream interferon-stimulated genes (ISG) to enhance the antiviral effect, such as OAS, Mx1, and STAT-1 [25,26,27,28].

In our previous study, canine RIG-I in MDCK cells played an essential role against canine influenza virus (CIV) infection [18]. Interestingly, an outcome of more than 2500 ng canine RIG-I overexpression showed a gradually decreasing antiviral effect against CIV, even though overexpressed canine RIG-I indeed suppressed the CIV replication in MDCK cells and HEK 293T cells [18]. Here, we performed the same experiments again, and the results indicated a similar trend to our previous study. This suggested the authenticity of the unknown negative regulations triggered by the increasing dose of RIG-I overexpression.

When the exogenous pathogens were exposed in intracellular detection of the innate immune system, cells were triggered to produce the antiviral response, even the harmful overreacted effects. For cellular homeostasis, cells would balance themselves and negatively regulate these excessive reactions by several biological systems. To date, many studies have indicated some negative regulations for RLRs signaling pathway, including regulation by ubiquitin ligases and deubiquitinases [29,30,31,32], kinases and phosphatases, host protein modifications [33], RNA-binding proteins, and viral proteins [34]. Recently, autophagy has been pointed out as new negative regulation of the RLRs signaling pathway [35,36,37,38,39]. These studies present a complex cross-reaction between the RLRs signaling pathway and the cellular autophagy.

To investigate whether autophagy was involved in our findings, we detected the level of LC3B protein [40,41] derived from samples with the same treatments as our previous study. The results showed that the ratio of LC3B-II/GAPDH was increased in higher expression of canine RIG-I against CIV. It suggested the probability of autophagy-related regulation to RIG-I in CIV infection. 

However, the proteins derived from the influenza A virus have been reported as the regulator of RIG-I by different mechanisms, including ubiquitination and the autophagy process. To exclude the influence of CIV proteins, we carried out further experiments to explore the factors related to autophagy without CIV infection. First, the autophagy flux was determined by fluorescence analysis in RIG-I overexpressing cells. We found that LC3 puncta were significantly raised, and P62 spots were significantly diminished, compared to the mock group. In addition, here, MG132 (which could promote the autophagy process in high concentration [42,43,44,45]) and rapamycin were used to induce autophagy; ly294002 and chloroquine were used to inhibit the different stages of autophagy. Results of the dual-luciferase assay and Western blotting showed the autophagy inducer and inhibitor could decrease and increase the expression of RIG-I, respectively. Taken together, these findings provided robust evidence of autophagy-related negative regulation.

Our previous study indicated that canine RIG-I shares similar function domains with human RIG-I as a homologous gene. Canine RIG-I could trigger the antiviral effect in the human cell line [18]. Therefore, we chose the HEK 293T cells with high transfection efficiency for understanding the comprehensive regulations in cells transfected with exogenous RIG-I and revealing the potential genes for autophagy-related negative regulation by transcriptome analysis. The transcriptional screening indicated 1666 differentially expressed genes (DEGs), including 1384 up-regulated and 282 down-regulated DEGs, respectively. The GO and KEGG signaling pathway enrichment analysis of DEGs provided a complex regulation network of exaggerated expression of RIG-I, including inflammation, catalytic activity regulation, the nervous system regulation, and the JAK-STAT signaling pathway. It would lead to a more comprehensive understanding of the regulation of excessive expression of RIG-I. After the enrichment analysis of DEGs, we focused on the ubiquitination-related and autophagy-related DEGs, which may serve as a complex regulation for the negative regulation of RIG-I. These DEGs in volcano plot visualization were validated by qPCR. PTPN22, UBA7, PRKN, SMAD7, TRIM72, and OTUD7B genes were significantly up-regulated. SIRT2 and BFAR genes were in significant down-regulation. Recent studies indicate the autophagy-related function of these genes. PTPN22 regulates NLRP3-mediated IL-1B by autophagy [46,47,48]. PRKN (Parkin) could negatively regulate the antiviral response by degradation of TRAF3 [29,49,50]. OTUD7B serves as a deubiquitinase of P62/SQSTM1 and enhances the degradation of IRF-3 [51,52]. SIRT2 dysregulates autophagy in high-fat-exposed immune-tolerant macrophages, and the knockdown of SIRT2 increases basal autophagy [53,54,55]. With the combined analysis of DEGs and reports mentioned above, our study indicated that PTPN22, PRKN, OTUD7B, and SIRT2 genes were strongly correlated to the negative regulation of excessive expression of RIG-I.

In conclusion, canine RIG-I was confirmed to trigger autophagy when activating RLRs signaling pathway. This present transcriptome analysis provided a more comprehensive network to understand the regulation of excessive expression of RIG-I; it also revealed the potential genes involved in the negative autophagy-related regulation for more future studies.

## Figures and Tables

**Table 1:** qPCR was used to verify the ubiquitination and autophagy-related DEGs.

Protein-Coding Gene Name	NGS (log_2_ Fold Change)	Regulation Style	qRT-PCR (2^−∆∆Ct^ Fold Change)
PTPN22	4.97	Up	2.31 *
UBA7	2.48	Up	2.97 *
RASD2	2.46	Up	1.26
PRKN	1.47	Up	2.32 *
SMAD7	1.31	Up	1.41 *
TRIM72	1.18	Up	1.67 *
OTUD7B	1.11	Up	2.01 *
SIRT2	−2.83	Down	−2.56 *
BFAR	−3.75	Down	−2.75 *

* Asterisk indicates the statistical significance of DEGs with *p* < 0.05.

## Data Availability

The data that support the findings of this study are available from the corresponding author upon reasonable request.

## Supplementary Materials

The following are available online at www.mdpi.com/article/10.3390/cells11132009/s1. Supplementary Table S1: plasmids used in this study. Supplementary Table S2: sequence of siRNA for canine RIG-I. Supplementary Table S3: antibodies used in this study. Supplementary Table S4: Primer pairs used for quantitative RT-PCR analysis. Supplementary excel file 1: All DEGs are listed in the excel file.
