# Supplementary material for: Transcriptome Analysis of Retinoic Acid-Inducible Gene I Overexpression Reveals the Potential Genes for Autophagy-Related Negative Regulation
Source: Cells. 2022 Jun 23;11(13):2009. doi: 10.3390/cells11132009 (PMC9265583; doi:10.3390/cells11132009)
Supplement: Supplementary file 1 [file cells-11-02009-s001.zip › New folder/Supplementary Table S1-4.pdf]

Table S1 Plasmids used in this study

Plasmid	Reference
p3xFLAG-N-CMV-10	doi.org/10.3390/v13102048
Flag-RIG-I	doi.org/10.3390/v13102048
LC3-GFP-mCherry	doi.org/10.1080/15548627.2020.1725375
pCMV-mCherry-p62	doi.org/10.1074/jbc.M702824200
IFN-β luciferase reporter	doi.org/10.3390/v13102048
NF-κB luciferase reporter	doi.org/10.3390/v13102048
pRL-TK luciferase reporter	doi.org/10.3390/v13102048

Table S2 Sequence of siRNA used for canine RIG-I

Primer	Sequence of oligonucleotides (5′ → 3)
psiRIG-I-2	TGAGTAAGTTACACCCTAA

Table S3 Antibodies used in this study

Antibody	Company	Host species	Catalog number	Dilution
RIG-I	Prepared by our lab	Rabbit	None	1:2000
Flag tag	GeneTex	Mouse	GTX82562	1:1000
IAV NP	GeneTex	Rabbit	GTX125989	1:1000
LC3B	Abcam	Rabbit	AB192890	1:2000
Alexa Fluor® 680	Abcam	Goat	AB175781	1:5000
Alexa Fluor® 790	Abcam	Goat	AB175781	1:5000

Table S4 Primer sets used for qPCR in this study

Primer	Sequence of oligonucleotides (5′ → 3)
PTPN22	ACAACTGTGGCTGAGAAGCCCA
	GTAGCTGGAATCCTCATCAGAGG
UBA7	GAACTCTTCCGACTGTCTGCAG
	AGTCTTGCCAGTTCTGTGGACG
RASD2	GACATCCTGGATACCTCTGGCA
	CGGTTATCCAGGCTGAACACCA
PRKN	CCAGAGGAAAGTCACCTGCGAA
	CTGAGGCTTCAAATACGGCACTG
SMAD7	TGTCCAGATGCTGTGCCTTCCT
	CTCGTCTTCTCCTCCCAGTATG
TRIM72	TGCGTAAGGAGAAGAGTGTGGC
	AGCCAGGAACACCCGCATCTTG
OTUD7B	TCTCAGAGGCTGCTTCCTTTGG
	CGCCTTTTCAACGCTTCCTTCTC
SIRT2	CTGCGGAACTTATTCTCCCAGAC
	CCACCAAACAGATGACTCTGCG
BFAR	AGGCTGAGTCTGCTCTACCTGT
	ACTGCTTCCACGTAGGCTCCTT
